# Particle Therapy for Breast Cancer

**DOI:** 10.3390/cancers14041066

**Published:** 2022-02-20

**Authors:** Roman O. Kowalchuk, Kimberly S. Corbin, Rachel B. Jimenez

**Affiliations:** 1 Department of Radiation Oncology, Mayo Clinic, Rochester, MN 55905, USA; kowalchuk.roman@mayo.edu (R.O.K.); corbin.kimberly@mayo.edu (K.S.C.); 2 Department of Radiation Oncology, Massachusetts General Hospital, Boston, MA 02114, USA

**Keywords:** protons, carbon, breast cancer, particle therapy

## Abstract

**Simple Summary:**

Approximately 60% of patients with breast cancer require radiotherapy. With significant advances in breast cancer outcomes, an increasing focus is being placed on cancer survivorship, including the reduction of the late effects from radiotherapy. The aim of this review article is to highlight the potential of particle therapy in ensuring comprehensive radiotherapy for patients with breast cancer while reducing normal tissue exposure that can lead to subsequent impairments in quality of life. We review the latest literature in this space for both proton and carbon therapy and highlight opportunities for future study.

**Abstract:**

Particle therapy has received increasing attention in the treatment of breast cancer due to its unique physical properties that may enhance patient quality of life and reduce the late effects of therapy. In this review, we will examine the rationale for the use of proton and carbon therapy in the treatment of breast cancer and highlight their potential for sparing normal tissue injury. We will discuss the early dosimetric and clinical studies that have been pursued to date in this domain before focusing on the remaining open questions limiting the widespread adoption of particle therapy.

## 1. Overview: Enhancing Outcomes for Breast Cancer Patents

Advances in multi-disciplinary care have led to improvement in overall outcomes for women diagnosed with breast cancer, with a 40% decline in breast cancer mortality seen over the last 28 years [1]. With improved outcomes, emphasis has shifted to enhanced survivorship. Clinicians and researchers are increasingly focused on understanding long-term treatment-related sequelae and investigating approaches that prioritize quality of life. Multi-disciplinary examples include the de-escalation of axillary surgery and the use of the 21-gene recurrence score to spare many women from adjuvant chemotherapy [2,3]. In radiotherapy, over the last decade, improved adoption of advanced planning techniques has yielded improvements in dose distributions across target volumes and reduced exposure to organs at risk (OAR). Among radiotherapy techniques, particle therapy has emerged as a particularly attractive therapeutic option, largely based on its unique physical properties which result in very little dose beyond the target. Compared with conventional or photon-based approaches, particle therapy results in lower radiation dose exposure to non-target tissue, often while offering improved target coverage. Potential adverse effects from breast radiotherapy are often related to radiation exposure of nearby non-target tissues with no therapeutic indication for radiation, such as the heart and lung. As such, particle therapy offers the potential to improve the therapeutic ratio of radiotherapy. In the following review, we will highlight the emerging role for and clinical use of particle therapy, particularly proton therapy, in the treatment of breast cancer by synthesizing all available literature in this space.

## 2. Proton Therapy: Rationale and Specific Clinical Indications

### 2.1. Rationale

**Figure 1:**
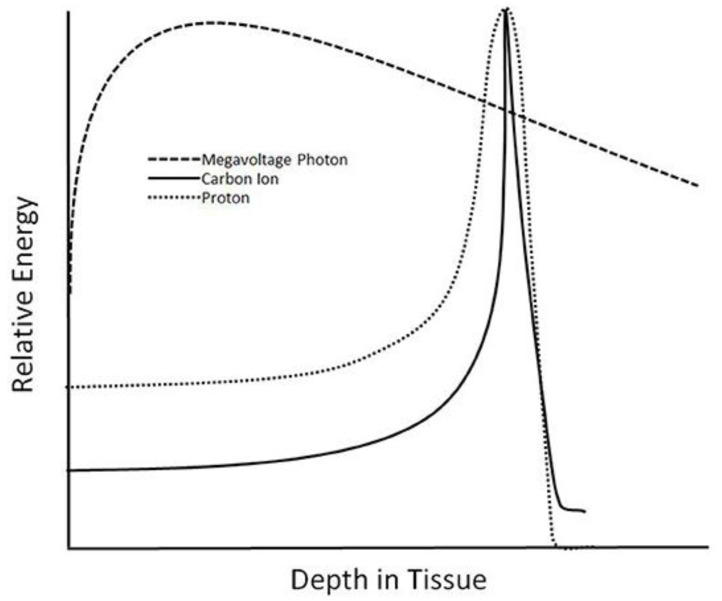
A comparison of the dose deposition in tissue is shown between radiation therapy using six MV photons, protons, and carbon ions [4].

Particle therapy, by virtue of its physical properties, can better spare normal tissue, or OARs, in proximity to the radiation target, compared to conventional radiation. Specifically, both proton therapy and carbon therapy exhibit a narrow area of high energy deposition with a sharp dose fall off, called a Bragg peak. This distribution of radiation, in contrast, to conventional X-rays, permits more precise dose shaping, particularly distal to the radiation beam, resulting in greater sparing of low dose to normal tissue beyond the target (Figure 1). In the treatment of breast cancer, the OARs that typically receive excess radiation include those organs located close to the breast and regional lymph nodes, particularly the heart and lungs.

### 2.2. Cardiac Dose

Much attention has been paid to the cardiac sparing properties of proton therapy given the well-described association between mean cardiac dose exposure and subsequent major cardiac events. In the oft-cited study by Darby and colleagues of greater than 2100 women with breast cancer receiving radiation therapy, a linear relationship was demonstrated between the risk of ischemic cardiac events and estimated mean radiation dose exposure to the heart, wherein each increase in 1 Gy of mean radiation dose exposure to the heart corresponded to a 7.4% increase in major coronary events (MCE), with no minimum threshold for events [5]. More modern dose modeling studies of cardiac radiation exposure, including that of Van den Bogaard and colleagues, utilizing 3-dimensional imaging, estimated an even more pronounced risk of acute coronary events, a 16.5% increase per 1 Gy in mean heart dose. Leveraging the use of CT-based planning, Van den Bogaard further analyzed the risk of events corresponding to dose exposure to cardiac substructures and showed that the volume of the left ventricle receiving 5 Gy (LV-V5) was a better predictor of MCE than mean heart dose alone [6]. Subsequent secondary analyses of the data reported by Darby and colleagues assessing coronary artery segments also showed that higher RT dose at any segment of the coronary vessels was associated with injury, suggesting that all anatomic components of both the left ventricle and coronary arteries could generate cardiac impairment [7]. Therefore, the global and approximately 10-fold reduction in cardiac and cardiac substructure dose exposure with proton therapy, as demonstrated in numerous retrospective and small prospective studies, suggests that proton therapy may be cardioprotective [8,9,10,11,12].

**Figure 2:**
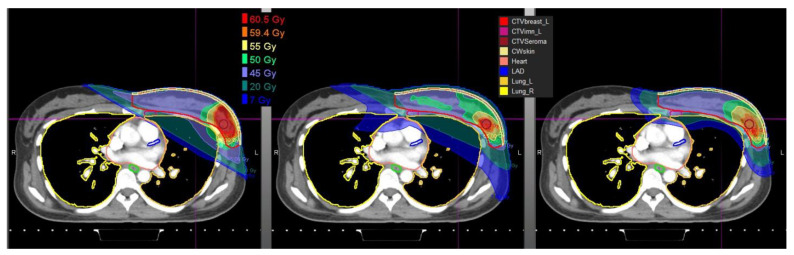
A comparison of the dose distribution comparing the same patient planned with 3D, VMAT, and pencil beam proton therapy.

Cardiac sparing holds particular value when considering target coverage to the internal mammary nodes (IMN). Coverage of the IMN with breast radiotherapy has been shown to improve overall and disease-free survival for women with high-risk breast cancer, both in a population-based trial and in the meta-analysis of the EORTC 22922 and MA.20 trials [13,14,15,16]. However, IMN irradiation is associated with increased cardiac exposure to radiation, thereby increasing the potential adverse effects of treatment [17]. While modern radiotherapy techniques have improved the ability to cover breast cancer targets, compromise of target volume coverage or excess doses to heart and lung are still frequent. In one striking example from Europe of modern radiotherapy cases, authors re-planned treatments without allowing target compromise. The estimated portion of breast cancer patients with excess doses to either the heart or the lung was 22%, suggesting that clinicians compromise target coverage in about 1 in 5 patients [18]. Particle therapy may be especially valuable among left-sided patients receiving regional nodal irradiation (RNI), wherein the IMN are a target structure and are located immediately adjacent to the heart, as well as in young women, and those with pre-existing cardiac risk factors [5,19]. Prospective studies do confirm significantly greater dosimetric advantages for proton therapy in the setting of RNI [20,21] (Figure 2), wherein comprehensive coverage of the IMN’s has been shown to be achievable without undue cardiac exposure [20,22].

When considering proton therapy’s cardiac sparing properties, some have also posed the advantage of combining other cardiac sparing approaches, including deep inspiration breathhold techniques (DIBH), with proton delivery with the goal of furthering limiting cardiac exposure. However, many institutions do not utilize DIBH with proton therapy, despite widespread use of DIBH with photon therapy because of the additional technical requirements of DIBH and because of the modest additional dosimetric benefits suggested when DIBH is paired with proton dosimetry [23]. However, no universal consensus exists and additional study may identify specific patients for whom combination DIBH and protons are beneficial.

### 2.3. Lung Dose

Proton therapy can also be effective at limiting both high- and low-dose exposure to the ipsilateral and contralateral lungs. Taking advantage of the steep dose fall-off of the Bragg peak, the dose gradient is sharp, allowing for reduction of lung tissue in the treatment field. While modern estimates of grade 2 or higher radiation pneumonitis (1–3%) and pulmonary fibrosis (4–5%) after photon-based RNI are modest [15,24], further dose sparing is possible with proton therapy. In a prospective study of proton beam radiation for patients receiving RNI promisingly low rates of pulmonary toxicity were reported, with only 1 patient with grade 2 pneumonitis and no instances of grade 3 radiation pneumonitis [22]. Further research is needed to identify those with the greatest potential advantage to pulmonary sparing, including both the sub-acute risk for pneumonitis, as well as delayed risk for fibrosis and lung cancer. Dose modeling studies of secondary lung cancer after radiation for breast cancer have shown significantly lower risks of radiation-induced pulmonary malignancy with the use of proton beam therapy compared to conventional three-dimensional or intensity-modulated radiation therapy [25,26]. This may be particularly important among patients who are current and former smokers, given their relatively higher risk of developing a secondary lung cancer (4% absolute risk for active smokers compared with 0.3% for non-smokers) [27,28].

### 2.4. Dose to the Intrinsic Muscles of the Shoulder and Chest

Musculoskeletal sparing including relative sparing of the intrinsic muscles of the shoulder has been posited as potentially beneficial to reduce arthritis and/or extremity symptoms. Upper limb disability is a common problem after breast cancer therapy [29]. Women undergoing surgery, including reconstructive surgery, and radiation often have musculoskeletal changes such as reduced shoulder range of motion, chest wall pain, lymphedema, and paresthesias [30]. These impairments can lead to a decline in quality of life and generate lasting disability and discomfort that can extend for years after completion of treatment [31]. Proton therapy for treatment of the regional nodes can result in lower doses to the intrinsic muscles of the rotator cuff, humeral head, and scapula. Though proton therapy improves tissue sparing, the role of proton therapy to improve shoulder/arm quality of life warrants further investigation, and correlation between dosimetric reductions and patient-reported outcomes is needed.

Importantly, as a consequence of pre-existing shoulder or arm conditions or as a result of axillary dissection, shoulder abduction and/or external rotation may be limited at the time of radiation planning. In these women, proton therapy (especially with the en-face field technique) can be used to spare normal tissues of the arm and shoulder while treating patients in an arms-down or akimbo position without limiting therapeutic doses to target tissues [32]. Photon-based radiation therapy planning with the arms down is technically challenging, with higher radiation doses expected to the upper limb with standard approaches.

### 2.5. Contralateral Breast

As proton beam therapy is not reliant on tangential fields to avoid normal tissue, the contralateral breast does not receive meaningful radiation exposure, resulting in a lower estimated risk of contralateral breast cancer, compared to other photon-based radiation techniques [12,25,33,34]. Such considerations for the contralateral breast and subsequent cancer risks may be of particular value for young women (age < 40 years), those who harbor heightened risk from deleterious mutations, or those with challenging anatomy [35].

### 2.6. Additional Anatomical Considerations

Geometric challenges may arise in those undergoing standard intact breast or post-mastectomy radiotherapy with photons for a number of reasons. For those undergoing mastectomy, reconstruction is increasingly pursued by women. Target coverage may be compromised, or doses to OARs may be higher, particularly among those with bilateral reconstruction with permanent implants [36,37] as they may be positioned in the path of the tangential beams.

**Figure 3:**
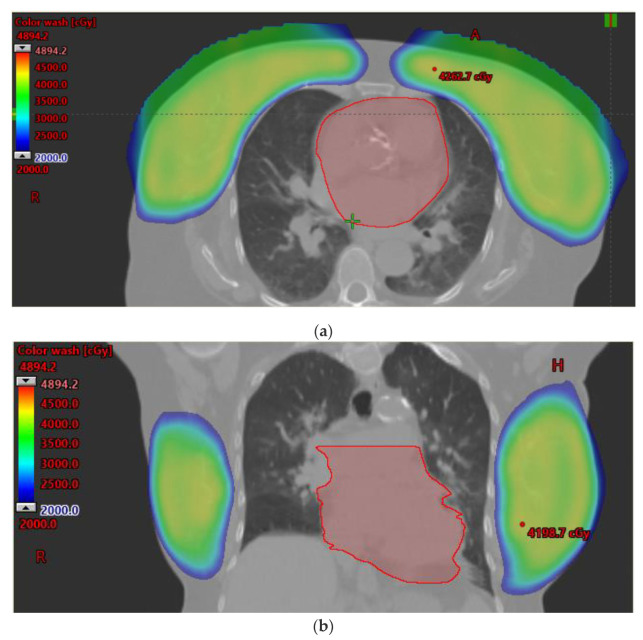
(**a**) An example of extensive target volume coverage is demonstrated. Treatment with proton therapy to a dose of 64 GyE was delivered. The mean heart dose was 0.44 GyE, with a volume receiving 5 GyE of 2.8%. The volume of right lung receiving 20 GyE was 13.3%, and the total volume of lung receiving 20 GyE was less than 10%. (**b**) A case involving bilateral breast cancer is shown. The target was defined as the bilateral whole breast, and a boost was planned within the left breast. The patient suffered from baseline pulmonary hypertension and congenital heart block, and she was not able to perform deep inspiratory breath hold. Despite these features, only 0.4% of the total lung received at least 20 GyE, and the mean heart dose was 0.3 GyE.

Other underlying anatomic variations can lead to considerable geometric challenges for photon radiation. For example, when larger than typical target volumes are required, such as those with inflammatory breast cancer, deep nodal boosts, or extensive skin involvement, photon radiotherapy target coverage may be compromised. Yet, these are often the scenarios in which such compromise could be most detrimental, and the recurrence risk highest, suggesting that improved target coverage using proton therapy may meaningfully reduce risk for recurrence (Figure 3a). In addition, in the setting of bilateral breast cancer, proton therapy offers a significant dosimetric advantage, as the larger target volumes increase OAR exposure to radiotherapy doses, particularly in the setting of reconstruction or regional nodal irradiation (Figure 3b) [38]. In one planning study for synchronous bilateral cancer, VMAT or hybrid plans resulted in mean heart exposures of 13.2 and 9.7 Gy, respectively [39], much higher than achievable with proton therapy. The estimated benefit is greater in those with underlying cardiac risk factors, as a result, the cardiac sparing potential may be of particular importance for those with advanced age or the presence of hypertension, diabetes, hypercholesterolemia, and smoking [6].

Pectus excavatum (PEx) also often results in anatomic distortion of the cardiac position, making appropriate cardiac sparing with typical photon approaches challenging. Compared with optimal VMAT-BH plans, proton therapy reduced mean heart dose, lung dose, and contralateral breast dose in a planning study of women with PEx [34]. Dosimetric improvements would be expected in other scenarios with unfavorable cardiac position, including those with significant cardiac chest contact. Finally, though the role for loco-regional therapy in the setting of oligometastatic breast cancer is not established, at least one large published series have shown improved outcomes with inclusion of local therapy to sites of contiguous metastatic involvement, such as the sternum or mediastinum [40]. However, inclusion of these sites can be technically challenging with photon radiotherapy due to the larger field size, and the use of proton therapy may allow for safe consolidation of such a patient’s disease [41].

In scenarios under which comprehensive target coverage is desired and particle therapy is utilized for breast cancer radiotherapy, some normal tissue exposure may be counterintuitively higher than when using three-dimensional conformal radiotherapy. For example, the medial portion of the supraclavicular fossa is typically omitted from the radiation field when using three-dimensional conformal radiotherapy due to concerns of spinal cord and spinal nerve root exposure. With particle therapy, more complete target coverage of the supraclavicular fossa can be achieved due to the precise dose shaping of the beam, but with the trade-off of slightly higher exposures to the esophagus and thyroid gland, similar to what can be observed with volumetric arc therapy, or other inverse planned photon techniques [25]. Therefore, it is vital that physicians determine the prioritization of target coverage versus normal tissue exposure during the planning process.

### 2.7. Situations of Greater Than Average Baseline Risk

**Figure 4:**
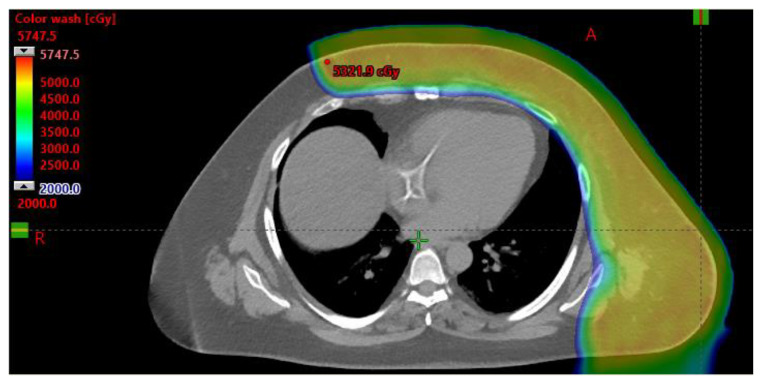
A case of pre-operative reirradiation is shown, involving the necessity for extensive tissue coverage. Radiotherapy was delivered for the indication of gross residual disease, and proton therapy allowed for delivery of treatment with maximal cardiopulmonary sparing.

The unique dosimetric advantages of proton therapy may offer particular benefit in scenarios in which reduction of normal tissue exposure is of heightened importance. For example, the risk for contralateral breast cancer is high among those with underlying genetic mutations [42]. Such patients may benefit from reduced dose to both the contralateral tissue and OARs, given the potential cumulative toxicity from a second course of therapy. Rare mutations can also carry enhanced sensitivity to radiation therapy. Germline mutations in TP53, for example, predispose not only to primary malignancy development, but also higher risk of treatment-related malignancy [43], with reports of up to a 33% risk of radiation-induced sarcoma in women undergoing breast radiotherapy [44]. While avoidance of radiation is often the strongest recommendation, in some very high-risk circumstances, radiotherapy is necessary and particle therapy can be utilized to reduce extraneous doses. In addition, in the setting of reirradiation, sparing of normal healthy tissues without compromising target coverage is often critical [45] (Figure 4). Finally, adolescent and young adult patients are widely accepted to have the most to gain from the reduced integral dose from particle therapy. These groups are the most vulnerable to the development of late radiation-related late toxicities, based on both heightened risk associated with young age at radiation exposure and expected decades of survivorship [46].

### 2.8. Reconstruction and Contouring Implications

For those undergoing reconstruction with tissue flaps, particle therapy offers the unique ability to dose paint, allowing for potential advantages such as sparing of the vascular anastomosis. This sharper dose fall-off could result in other anatomic regions outside of historically delineated CTVs receiving a lower dose than what was delivered with photon-based treatments; however, whether or not this reduction results in a clinically meaningful recurrence risk in these regions remains unknown. For example, intentional sparing of the dissected levels I/II when deemed appropriate has been associated with lower risk for lymphedema, and proton therapy could further enhance sparing of the dissected axilla. Further investigations into defining the highest risk regions may yield opportunities for customization or dose painting within the CTV, as the updated ESTRO contouring guidelines suggest that deep and lateral tissue may be spared [47].

There are presently few data that report on reconstructive results following radiotherapy with particles; however, early findings have been encouraging. In a prospective trial of 69 patients treated with proton therapy, 83% pursued breast reconstruction and while approximately one-third experienced some form of unplanned surgical re-intervention at 5 years, only 4% experienced reconstructive loss attributable to radiation [22]. Importantly, the use of proton therapy for breast cancer remains a critical topic for study, and reports of long-term cosmetic outcomes, with a focus on skin dose and fractionation, will be required to further refine treatment planning and decision-making.

## 3. Hypofractionation

Moderately hypofractionated radiotherapy, using doses of approximately 2.65–2.67 Gy per day, is established as the current standard of care for women undergoing adjuvant whole breast radiotherapy, based on equivalent oncologic outcomes, with similar or reduced toxicity reported in long term follow-up [48,49]. Hypofractionation using proton therapy is an active area of exploration in the setting of clinical trials. The Mayo Clinic MC1631 trial comparing 15 versus 25 fractions for 88 women undergoing post-mastectomy radiotherapy with or without reconstruction completed accrual and is pending publication. Additional multi-institutional clinical trials in the United Kingdom and Denmark using moderate hypofractionation with proton therapy for the treatment of regional lymph nodes will further refine the role of proton therapy in this setting [18,50].

The use of ultrahypofractionated whole breast and regional nodal irradiation, using 5.2–5.7 Gy per day, is also receiving increasing attention based on equivalent oncologic outcomes with similar toxicity reported in short-term follow-up from the UK FAST FORWARD trial [51]. One clinical trial, MC1635, comparing 5 versus 15 fractions for 107 women treated with breast-conserving surgery, has reported initial follow up and 50% of women in that study were treated with proton therapy, with just 1 patient treated with short course therapy experiencing cosmetic deterioration [52]. Additional prospective data is needed in this context to provide needed information regarding efficacy and toxicity.

## 4. Partial Breast Irradiation

Accelerated partial breast radiotherapy (APBI) has emerged as an appropriate form of adjuvant radiotherapy following lumpectomy in women with favorable early-stage breast cancer, such as those with small, hormone receptor positive, lymph node negative disease. The data have consistently demonstrated that, in select populations, APBI enables more convenient, efficient therapy with similar oncologic outcomes [53,54,55,56,57,58,59,60]. Variation in patient selection, technique, target volume, dose and fractionation may influence expected results from an oncologic and cosmetic perspective. Nevertheless, the volume of non-target breast tissue exposed to radiation appears to be correlated with the risk for adverse cosmesis [61,62]. Proton therapy offers a distinct advantage in reduction of the volume of normal breast tissue exposed to radiation and may offer some advantages to limiting exposure to the contralateral breast and OARs for women with an unfavorably situated seroma, or those with a larger volume of non-target breast tissue exposure [35]. APBI also allows for high doses of radiation to be delivered per fraction, resulting in accelerated therapy to maximize patient convenience and minimize associated travel and financial burdens. While limited initial reports described high rates of fibrosis, telangiectasia, and impaired cosmesis with proton APBI, this was possibly related to the use of a single passively scattered proton field, which risks increased dose to the non-target tissue in the path of the beam [63]. A larger experience with 5 years of follow-up using a two-field approach found more favorable oncologic and cosmetic results [64]. Similarly, a 100 patient cohort phase II trial was recently reported demonstrating high local control, patient satisfaction, low treatment burden, and minimal dose to the heart and lung [65]. Finally, a 3-fraction regimen of proton-based APBI, reported good or excellent cosmesis in 95% of patients, but additional study is needed [66].

## 5. Carbon Ion Therapy

Radiotherapy techniques utilizing carbon ions offer many similar physical advantages to those achieved using proton therapy, albeit with less prospective literature to date to provide support for use in breast cancer [4] (Table 1). The limited clinical experience reflects a relative scarcity of carbon therapy facilities, with at least 12 facilities worldwide [67]. Nonetheless, compelling data in a range of oncologic settings (including bone and soft-tissue sarcoma of the skull base, head and neck, and pelvis) from the last 20 years has spurred encouragement for further study [68]. As with proton therapy, carbon ion therapy also demonstrates a characteristic “Bragg Peak,” with sharper peak and narrower penumbra (Figure 1). These properties allow for the sparing of OARs both proximal and distal to the target. Additionally, carbon ion therapy involves a higher relative biological effectiveness (RBE) secondary to higher linear energy transfer (LET), by virtue of dose, beam energy, and cell type [69]. These properties offer the potential for improved cell kill while simultaneously minimizing toxicity due to the sharp Bragg Peak.

With the goal of harmonizing the unique characteristics of carbon ion therapy, a few studies have begun to demonstrate encouraging findings across diverse disease sites. For instance, the COSMIC study prospectively explored a combination of carbon ion therapy (24 Gy RBE) followed by 50 Gy IMRT for patients with malignant salivary gland tumors, with an encouraging three-year local control of 81.9%, and moderate acute and late toxicity [72]. Other, retrospective analyses of patients treated with carbon ion radiotherapy for skull base chordomas and primary lung cancers, have reported similarly promising cancer control and toxicity outcomes [73,74,75].

In breast cancer, utilization of carbon ion therapy is nascent, but has shown potential utility in early clinical case reports [76]. For instance, carbon ion therapy presents the potential for comparable secondary malignancy reduction to that seen with proton therapy [77]. This benefit is chiefly derived from the decreased integral dose of radiation delivered during the course of treatment. Much as with proton therapy, carbon ion radiotherapy may also facilitate ultrahypofractionation [76]. A recent phase I study seeking the optimal dose for a 4-fraction definitive carbon ion treatment regimen reinforced the safety of this approach [71,78]. In this trial, dose escalation up to 60 Gy (RBE) was delivered in 7 patients, and after at least 3 years of follow-up for each case, all patients were alive without disease or late adverse reactions.

Unfortunately, cost and resource barriers prevent widespread use of carbon ion radiation therapy for breast cancer. For example, while carbon ion treatment centers are open and treating patients in at least five countries, no carbon ion center has yet been built in the United States. In addition, the optimal clinical scenarios to justify the use of carbon therapy are not fully understood. Combining carbon ion radiation therapy with existing modalities, as in the COSMIC trial, could offer a novel approach to explore its utility, but for the reasons above, utilization is likely to progress slowly. Alternatively, carbon ion therapy may have a potential role involving definitive radiotherapy for breast cancer, as opposed to adjuvant therapy. However, such a substantial paradigm shift would also require further study.

## 6. Treatment Delivery Considerations

As particle therapy offers more precise dose shaping and treatment delivery, it is inherently more sensitive to changes in immobilization and daily set-up variations. This is true among women with reconstructed breasts because the implant and/or tissue expander can shift in position during treatment. It can also be identified in women with intact breasts who experience inflammatory changes and edema over the course of treatment. Consequently, daily set-up surface imaging in combination with X-ray and cone-beam CT is typically employed and in circumstances where shifts exceed institutional tolerance based on robustness calculations, it should prompt re-planning to ensure fidelity of the intended dose delivery [79,80].

## 7. Potential Biologic Advantages

While the central advantage of particle therapy has focused on its beneficial physical properties, there is emerging data to indicate that particle therapy may also hold unique biologic properties with implications for both normal tissue toxicity and cancer control. For example, while it is assumed that the average radiobiologic equivalent dose (RBE) of protons relative to photon beams is approximately 1.1, it has been supported through multiple studies that the acute RBE values may be higher, particularly at the end of range [81,82]. Normal tissues immediately distal to target structures, such as the ribs for breast cancer patients, may therefore receive a higher biologic equivalent dose than is intended, raising the risk of rib fractures [83]. In addition, among cancer cells with defects in homologous recombination, including BRCA-related malignancies, and Fanconi Anemia pathways, observed RBE values can be high as 1.3–1.5 in-vitro, suggesting that these malignancies may be more sensitive to proton therapy than conventional radiation [84,85,86,87]. Building upon these observations, there have been limited in-vitro studies of combination particle therapy with both PARP-inhibitors and cisplatin chemotherapy that have demonstrated enhanced breast cancer sensitivity with the combination of systemic therapy and particle beam radiation versus systemic therapy and conventional X-ray radiation [88,89,90]. Carbon therapy also exhibits a variable RBE exceeding that of both photon and proton therapy and offers an opportunity for the successful treatment of breast cancer in specific clinical scenarios. For instance, the increased clustering of multiple forms of DNA damage may be particularly beneficial in treating triple-negative breast cancer [90]. Other potential applications include the metastatic setting (with local therapy providing activating effects for the immune system) and/or in the treatment of breast angiosarcomas [91]. While the promise of personalizing particle therapy for those with radioresistant tumor types remains compelling, high-level evidence is lacking to date and additional study is both ongoing and warranted.

## 8. Cost-Effectiveness

Despite the rapid increase in indications for, and the availability of, particle therapy across the world, it remains a limited resource [92]. Accordingly, to ensure effective and equitable care, optimization of patient selection is required. One analysis used a Markov cohort model to compare the cost-effectiveness of proton versus photon radiotherapy for breast cancer management for patients with and without coronary heart disease [93]. Using a standard willingness-to-pay threshold of $100,000 per quality-adjusted life-year, proton therapy was not cost-effective for women without cardiac risk factors or with a mean heart dose of less than 5 Gy. For women with at least one cardiac risk factor and a mean heart dose of at least 5 Gy, proton therapy was cost-effective. Although the likelihood of cardiac events is low, the costs when they do occur are substantial. As a result, studying the cost-effectiveness of proton therapy by focusing on the reduction in major cardiac events is likely a reasonable, though imperfect approach. Presumably, a threshold reduction in major cardiac events exists above which proton therapy would consistently demonstrate cost-effectiveness, but the derivation of this threshold is complex [94]. While intriguing, these results may underestimate the cost-effectiveness of proton therapy by not considering other benefits of proton therapy, such as the reduction in secondary malignancies.

Future comprehensive cost-effectiveness analysis could also consider individual risk profiles. Separate cost analyses may be warranted for additional scenarios in breast cancer radiotherapy, including those with altered fractionation. For instance, partial breast irradiation has historically been offered for carefully-selected elderly patients with favorable tumor characteristics; however, providing a 3-fraction proton-based PBI treatment may also provide cost-effective treatment to a young patient by minimizing secondary malignancies and cardiac risk [66]. Despite rigorous efforts, a modern, overarching cost-effectiveness analysis incorporating changes in the duration of treatment (e.g., hypofractionation) is still needed. It also may be that future technological advancements resulting in cost reduction of equipment may eventually lead to cost-effectiveness for proton therapy. Overall, though the cost-effectiveness window for proton therapy may be narrow, current utilization rates remain modest, and additional study is warranted [95].

## 9. Potential Pitfalls and Disadvantages

Exploring the full utility of particle therapy for breast cancer is still required in many domains. Suboptimal patient selection is a potential concern, as particle therapy may be maximally beneficial only in specific clinical situations. Further, particle therapy remains a limited resource not available to all patients, so judicious use is required for equitable healthcare [96,97]. Particle therapy is also associated with longer treatment times than photon-based treatments, increasing both the cost of production and the duration of therapy and requiring detailed analyses to maintain departmental treatment efficiency [98]. Finally, technical challenges remain for providers, including consideration of robustness and end of range effects (and range uncertainty), as well as variations in increased LET and RBE at the distal edge of proton beams, which may result in increased toxicity [83]. Robustness calculations are a critical component of treatment planning with particle therapy, but wide variations exist among institutional practices, and specific technical considerations of robustness are dependent on the specifications of each particle therapy machine and technique, which is beyond the scope of this review. While providers’ comfort and expertise will improve with time and experience, rigorous and careful study is imperative.

## 10. Conclusions and Future Directions

Particle therapy offers a radiobiologically distinct modality for the delivery of radiotherapy, and its use may improve patient outcomes and minimize toxicity in specific clinical situations in breast cancer. Future research is required to optimize patient selection for particle therapy [99]. We await further work to refine the clinical applications of particle therapy.

## Figures and Tables

**Table 1:** Published prospective studies of proton-based and carbon ion-based radiotherapy in the setting of breast cancer are tabulated below.

Study	Population	Key Findings	Reference
Prospective dosimetric study of regional nodal irradiation	Eighteen women requiring RNI	Proton therapy was used in 10 patients with mixed photon-proton plans in 8 patients; proton therapy improved coverage of level 2 axilla, IMN; median cardiac V5 and ipsilateral lung V5 and V20 were reduced	[20]
Phase II Regional nodal irradiation	Seventy patients requiring post-operative radiotherapy to breast-chest wall with RNI	Median 49.7 GyE to chest wall/breast and median 48.8 GyE to IMN. At a median follow-up of 55 months, 5-year locoregional failure 1.5% and OS 91%. One case of G2 RP; no grade 3+RP; no G4+ toxicity	[22]
Phase I proton APBI	Ninety-eight women with stage I breast cancer	19/98 patients received proton therapy (32 GyE in 8 fractions); good to excellent cosmesis in 62% of proton therapy patients at 7 years, 11% local failure	[35,63]
Phase II proton APBI	One hundred women with nonlobular carcinoma and maximal tumor dimension of 3 cm and negative axillary lymph nodes	At a median 60 months of follow-up, there was 94% disease-free survival and 95% overall survival; 90% good to excellent patient-and physician-reported cosmesis	[64]
Phase II proton APBI	One hundred patients with pTis or pT1-2N0 (≤3 cm)	Thirty-four GyE in 10 fractions. At a median 24 months of follow-up, no G3+ toxicity with excellent or good cosmesis at 12 months of 91% and 94%	[65]
3 fraction proton APBI	Seventy-six women with age ≥ 5 years, ER+, sentinel lymph node negative invasive or in situ ≤ 2.5 cm	21.9 GyE in 3 fractions; at median 12 months of follow-up, no G2+ toxicity observed; 98% of patients reported excellent or good cosmesis	[66]
Phase II proton APBI	Thirty women, pathologically negative axillary lymph nodes, maximal tumor dimension of 3 cm	30 GyE in 6 fractions; at a median 59 months of follow-up, no ipsilateral breast recurrences, all patients alive; 80% good to excellent cosmetic outcomes at 2 months, 69% at 3 years	[70]
Phase I carbon ion APBI	Seven patients with low-risk stage I breast cancer treated with definitive intent	Dose escalation up to 60 GyE controlled disease for all patients at ≥3 years of follow-up, with no G2+ toxicity reported	[71]

Abbreviations: RNI = regional nodal irradiation; RBE = relative biological effectiveness; IMN = internal mammary lymph nodes; OS = overall survival; RP = radiation pneumonitis; G2 = grade 2; APBI = accelerated partial breast irradiation; ER+ = estrogen receptor positive; V5 = volume receiving at least 5 Gy; V20 = volume receiving at least 20 Gy.
